# A serological survey of brucellosis in wildlife in four major National Parks of Uganda

**DOI:** 10.1186/s12917-021-02782-4

**Published:** 2021-03-01

**Authors:** Robert Aruho, Ewan T. MacLeod, Leonard Manirakiza, Innocent B. Rwego

**Affiliations:** 1 Uganda Wildlife Authority (UWA) Headquarters, Plot 7, Kira Road, Kamwokya, P. O Box 3530, Kampala, Uganda; 2 Division of Infection and Pathway Medicine, 1 George Square, Biomedical Sciences, Edinburgh Medical School, College of Medicine and Veterinary Medicine, The University of Edinburgh, Edinburgh, EH8 9JZ UK; 3 National Pharmacovigilance Centre, Uganda National Drug Authority, Ministry of Health, Kampala, Uganda; 4 Department of Biosecurity Ecosystem and Veterinary Public Health, Africa One Health University Network (AFROHUN), College of Veterinary Medicine, Animal Resources and Biosecurity (COVAB), Makerere University, Box 7062, Kampala, Uganda; 5 Department of Veterinary Population Medicine, One Health Division, College of Veterinary Medicine, University of Minnesota, St. Paul, MN USA

**Keywords:** Infectious diseases, Zoonoses, Buffaloes, Giraffe, Zebra, Lions, Elephants

## Abstract

**Background:**

Brucellosis is a contagious zoonotic disease of great public health and economic significance especially in developing countries. The disease affects humans and several species of livestock and wildlife. Studies on Brucellosis in wildlife in Uganda have been limited to single populations particularly in Queen Elizabeth National Park. This study aimed at estimating the percentage of positive samples of *Brucella* spp. in wildlife in four major national parks of Uganda. This was a retrospective survey which utilized archived samples collected from wildlife during the annual disease surveillance activities between 2013 and 2017.

**Results:**

A total of 241 samples from seven species namely African buffalo (*Syncerus caffer*, *n* = 109), African elephant (*Loxodonta africana*, *n* = 22), giraffe (*Giraffa camelopardalis rothschildi*, *n* = 41), Uganda kob (*Kobus kob thomasi*, *n* = 36), lion (*Panthera leo*, *n* = 6), plain zebra (*Equus quagga*, *n* = 25), and bushbuck (*Tragelaphus scriptus*, *n* = 2), were tested for antibodies using the Rose Bengal Plate Test. The overall percentage of positive samples in the four national parks was 31.1% (75/241; 95% CI: 25.6–37.2). Kidepo Valley National Park had a significantly higher percentage of positive samples of 55.9% (19/34; 95% CI: 39.5–71.1) compared to other sampled national parks (*p* < 0.05). Lions had significantly higher percentage of positive samples at 66.7% (4/6) than African buffalo at 48.6% (53/109, *p* < 0.0001). There were no antibodies for *Brucella* spp. detected in African elephant and bushbuck.

**Conclusion:**

This study shows variations in percentage of positive samples with *Brucella* spp. between species and across national parks and notably a high percentage with *Brucella* spp. in wildlife in Uganda than that recorded elsewhere in sub-Saharan region of Africa. Potential for transmission to other wildlife and spill over to livestock is high especially in national parks with high livestock-wildlife interaction.

## Background

Globally, brucellosis ranks among the top ten diseases at the wildlife-livestock interface and affects a wide range of species of wildlife. Cases of human and animal brucellosis have been recorded on almost all the continents [1]. Many scholars argue that wildlife are potential reservoirs for brucellosis and a potential source of infection to livestock. However, the role of wildlife in the epidemiology of brucellosis is not clear. A study conducted in Spain showed that when brucellosis is reduced in the livestock, it is also reduced in wildlife, implying that wildlife may not be actual reservoirs of infection [2].

Currently, there is a great concern about emerging diseases at wildlife-livestock interfaces. Research shows that 70% of emerging zoonotic diseases originate from wildlife [3], for example, Ebola, Marburg and recently Zika virus [4–7]. It is increasingly clear there is need to generate more information on important but neglected zoonotic diseases such as brucellosis in Uganda. An assessment by International Livestock and Research Institute (ILRI) [8] has identified brucellosis among the top 13 zoonoses that highly impact the poor communities in sub-Saharan Africa. This assessment identified Uganda, among other countries, with a high burden of brucellosis.

Across sub-Saharan Africa, brucellosis is highly prevalent in both wildlife and livestock [9]. A study by Waghela and Karstad [10] in Masai Mara wildlife reserve in Kenya found the prevalence of brucellosis of 18% and 31% in wildebeest and African buffaloes respectively. In a study conducted across five game parks in Zimbabwe, a seroprevalence rate of 17% for brucellosis was found in buffaloes [11]. The same study showed that seropositivity was higher in wildlife sampled at the interface with livestock. Assenga et al [12] and Waghela and Karstad [10] predicted that there could be transmission at the interface in such scenarios. There is a risk of *Brucella* transmission from wildlife to humans in sub-Saharan Africa due to bushmeat acquisition and consumption. For instance, it was found that bushmeat is a potential source of brucellosis for humans and that buffalo meat is the preferred source of bushmeat in Botswana [13]. With seroprevalence in buffaloes being 6% in Botswana, this could be a great risk to humans [13].

A few studies conducted in Uganda on brucellosis have concentrated on studying infection in livestock and humans [14–18]. To our knowledge, there have not been any studies published from national parks and wildlife areas in Uganda except for a single study by Kalema-Zikusoka et al. [19]. This study estimated the prevalence of brucellosis in African buffaloes in Queen Elizabeth National Park to be 2%. Studies conducted around Lake Mburo National Park have indicated a high seropositivity of 55.6% and 31.8% in cattle and humans respectively [14, 20]. Unlike in southern African countries where hunting for wild game is legal under certain circumstances, in Uganda bushmeat acquisition and consumption is illegal [21]. Despite strong law enforcement mechanisms, sometimes poachers succeed in their hunt for bushmeat which they then distribute through the ‘black markets’ along major transit routes disguised as livestock meat [22]. The illegality of acquisition and informal entry into the human food chains does not allow public health inspection of bushmeat in Uganda. The wildlife-livestock interface has been expanding as people continue to settle near wildlife protected areas in search of fresh water, pasture for livestock and fertile soils to support food production. According to the study by Godfroid et al. [23] such close wildlife-livestock interfaces provide potential opportunities for transmission and persistence of infection of brucellosis in populations. Brucellosis is endemic throughout the country with individual animal prevalence of 15.8% in south western Uganda, 5.1% in central Uganda, and 7.5% in northern Uganda [17, 20, 24].

The increasing human populations and concomitant insatiable demand for food has caused tremendous changes in husbandry such as intensification of agriculture [25]. The quest for more arable land for large scale commercial farming is pushing people and livestock closer to wildlife protected areas in Uganda. At the park boundaries, there is a mix of wildlife, livestock and humans as they compete for scarce resources. Sharing of resources such as food, grazing land, and water at the same time between wildlife, livestock and humans has become common resulting in human-wildlife conflict and an opportunity for disease transmission and spread of zoonotic diseases such as *Brucella* spp. [26]. Despite presence of facilities and expertise to study Brucellosis in Uganda, no studies have been conducted in wildlife involving more than one national park. This study was, therefore, undertaken to estimate the percentage of positive samples with *Brucella* spp. in wildlife in four major national parks of Uganda.

## Results

A total of 241 wildlife samples from four selected national parks of Uganda were analyzed for *Brucella* positivity. Wildlife samples analyzed came from buffaloes (*n* = 109, 45.2%), bushbucks (*n* = 2, 0.8%), elephants (*n* = 22, 9.1%), giraffe (*n* = 41, 17%), lions (*n* = 6, 2.5%), Uganda kob (*n* = 36, 14.9%) and zebras (*n* = 25, 10.4%).

### Percentage of *Brucella* positive samples at national park level

**Fig. 1:**
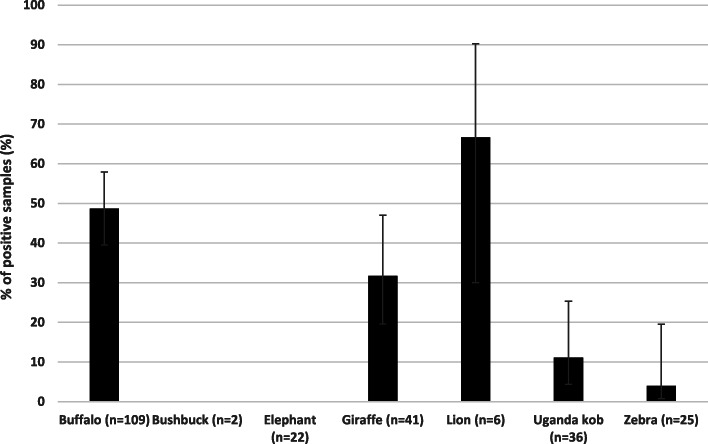
Percentage of *Brucella* positive samples in different wildlife species in selected Ugandan National parks

**Fig. 2:**
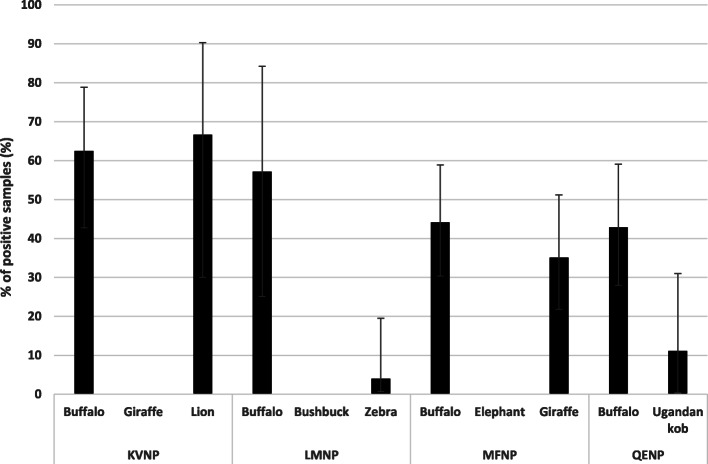
Overall *Brucella* seropositivity in wildlife species in the selected national parks of Uganda

Lions showed the highest overall percentage of positive samples of 66.7% (Fig. 1), although they were only sampled from Kidepo Valley National Park (Fig. 2). Buffaloes showed second highest percentage of positive samples (48.6%) overall, and with the exception of lions in Kidepo Valley National Park, showed the highest percentage of *Brucella* positive samples in each of the other national parks (Fig. 2). The overall percentage of positive samples in the four national parks was 31.1% (75/241; 95% CI: 25.6–37.2). Percentage of positive samples at national park level showed Kidepo Valley National Park had the highest percentage and Lake Mburo National Park had the lowest (Table 1). In addition, Kidepo Valley National Park had a significantly higher percentage of brucellosis compared to all other national parks (*p* < 0.05).

**Table 1:** Percentage of *Brucella* spp. Positive Samples in Four Major National Parks of Uganda

National Parks	No. of samples tested per National Park	% of positive samples per National Park	95% CI	Inter-Park comparisons	Z-Statistic	*P*-Value
				Comparison Park		
KVNP	34	55.9	39.5–71.1	LMNP	6.78	< 0.0001**
				QENP	3.83	0.0001**
				MFNP	3.08	0.001**
LMNP	34	14.7	6.5–30.1	QENP	−1.59	0.944
				MFNP	−2.10	0.982
				KVNP	−4.84	< 0.0001**
QENP	71	26.8	17.9–38.1	MFNP	−0.835	0.798
				KVNP	−4.94	< 0.0001**
MFNP	102	31.4	23.2–40.9	KVNP	−4.98	< 0.0001**

*KVNP* Kidepo Valley National Park, *LMNP* Lake Mburo National Park, *QENP* Queen Elizabeth National Park, *MFNP* Murchison Falls National Park

Overall, there was significant differences in percentage of positive samples in the national parks (G-Statistic = − 0.495, *p* < 0.0001). The percentage positive samples in Lake Mburo National Park was significantly lower than that of Kidepo Valley National Park (*p* < 0.0001; Table 1). In addition, percentage of brucellosis in Kidepo Valley National Park was significantly higher than that in the other three national parks (Table 1).

There were differences in percentage of positive samples of *Brucella* among the different animal species that were tested (G-statistic = − 0.659, *p* < 0.0001). The percentage of positive samples of *Brucella* in lions was significantly higher than that in buffaloes (*p* < 0.0001). The percentage of positive samples of *Brucella* in zebras was significantly lower from that of buffaloes (*p* < 0.0001), and lions (*p* < 0.0001) and giraffe (*p* < 0.0015, Table 2). The percentage of positive samples in Uganda kob was significantly lower than that from buffaloes (*p* = 0.001), lions (*p* = 0.003) and giraffe (*p* = 0.004).

**Table 2:** Inter-species Brucellosis Multiple Comparison

Base species (*Pr* = % of +ve samples)	Comparison species	% of positive samples	G-Statistic	*p*-value
Zebra (Pr* = 4.0)	Uganda Kob	11.1	−1.13	0.87
	Bush buck	0.0	–	–
	Buffaloes	48.6	−4.46	< 0.0001**
	Lion	66.7	−6.65	< 0.0001**
	Giraffes	31.7	−2.98	0.0015**
	Elephant	0.0	–	–
Uganda Kob (*Pr* = 11.1)	Bush buck	0.0	–	–
	Buffaloes	48.6	−4.80	< 0.0001**
	Lion	66.7	−7.08	< 0.0001**
	Giraffes	31.7	−2.66	0.004**
	Elephant	0.0	–	–
Buffaloes (*Pr* = 48.6)	Lion	66.7	−4.01	< 0.0001**
	Giraffes	31.7	3.79	0.0001**
	Elephant	0.0	–	–
Lion (*Pr* = 66.7)	Giraffes	31.7	1.84	0.033**
	Elephant	0.0	–	–
Giraffe (*Pr* = 31.7)	Elephant	0.0	–	–

*Pr =* % of positive samples; *+ve =* Positive

## Discussion

This was the first study looking at percentage of *Brucella* spp. positive samples in wildlife involving more than one national park in Uganda. In this study, the overall percentage of positive *Brucella* samples was 31.1% in the four major national parks. This is higher than the percentage of 2% recorded in wildlife in Queen Elizabeth National Park [19]. The percentage in wildlife in this current study is also higher than that recorded in livestock in Uganda [17, 20]. What is interesting in this study is that parks such as Kidepo Valley National Park, and Murchison Falls National Park that have relatively low livestock-wildlife interaction had far higher percentage of positive samples than Lake Mburo and Queen Elizabeth National Parks which have a very close livestock-wildlife interaction.

Among the national parks studied, Kidepo Valley National Park had the highest percentage of positive samples at 55.9%. This percentage is higher than the 9.2% recorded in cattle in Karamoja where the national park is situated [27]. It is not clear what the source of brucellosis in Kidepo National Park may be. The park is located in north east Uganda, a very remote area that has been characterized by insecurity for a long time [28]. There is less information available on the disease burden for the region. The veterinary extension services in the region have been almost non-existent with people relying on ethno-medicine to control cattle diseases [29]. Frequent cattle incursion in the park, especially during long dry spells, in search of water and pasture is a big opportunity for sustained infection in wildlife and cattle. It is therefore not surprising to find that a disease like brucellosis may have found a suitable niche. According Serrano et al. [2], brucellosis is well maintained in wildlife when interventions to control the disease in livestock are poor. Areas around Kidepo Valley and Murchison Falls National Parks have been recovering from the effects of Lords Resistance Army war which hindered agricultural extension services delivery in the region [30]. Although the percentage of positive samples in wildlife is higher than in cattle in the areas surrounding these national parks [17, 20], the direction of spread of brucellosis across wildlife and livestock is not clear and needs to be investigated. We did not detect any positives in the elephant or bushbuck samples, this agrees with previous studies that have not detected brucellosis in these animals [13].

The buffaloes in the four national parks sampled in Uganda had high percentage of positive samples of 48.6% compared to the 2% previously reported by Kalema-Zikusoka et al. [19] in Queen Elizabeth National Park. The rise in percentage of positive samples could be due to increased interactions with cattle infected with brucellosis at the wildlife interface. However, as percentage of positive samples did not vary much between the four national parks, and there were differences between the national parks in terms of cattle interaction, this could suggest that buffaloes play a role as a reservoir species. These findings are consistent with results from other studies conducted elsewhere in east and southern Africa by Motsi et al. [11], Alexander et al. [13], and Waghela and Karstad [10] that showed a higher percentage in wildlife. It is believed that buffaloes harbor *Brucella* better than other species for reasons not well understood [11]. Buffaloes are gregarious animals and usually live in big herds. Herd size has a big effect on the transmission of brucellosis [31]. According to Dobson and Meagher [31], brucellosis is well maintained in herd sizes of greater than 200 individual animals per herd. The disease prevalence tends to be high in big herds because the small inter-animal distance helps to sustain transmission by contact [32]. Therefore, herd sizes like those in Kidepo National Park (around 6900 buffaloes) are likely to maintain infection for a very long time without showing any impact on the population.

Four out of six lions sampled were positive. This was the highest percentage of positive samples of all the wildlife in this study. However, it is difficult to conclude if this is representative of *Brucella* infection in lions due to the small sample size tested in the current study. They have been few previous studies investigating *Brucella* seropositivity in lions. However, a study in Tanzania did find one positive lion out of two tested [12]. During field sample collection for the current study, one typical clinical case of brucellosis in lions was encountered. The affected lion had hygroma around joints and was always reluctant to move (Robert Aruho, *Personal observation*). This lion was positive for *Brucella* spp in this study. From the observations in the field, lions usually choose prey on which they will not spend a lot of energy to hunt. Clinically, sick animals affected by brucellosis usually develop mobility challenges because of dysfunctional joints and usually tend to move behind the herds. This makes the animals, such as buffaloes, easy prey by predators especially the lions which thrive best at hunting solitary prey [33]. Lions might also seroconvert due to exposure to *Brucella* through feeding on such infected animals. Previous work had shown that lions may become immune to *Trypanosoma brucei* *rhodesiense* infections due to being exposed to parasites through consumption of infected meat [34]. Such a scenario could be responsible for high percentage of *Brucella* spp. positive samples in lions of Kidepo Valley National Park. According to Uganda Wildlife Authority in 2018, the lion population in Kidepo Valley National Park was about 132 individuals. We tested a few individuals compared to the population size. Therefore, this calls for more studies to be undertaken in this lion population of Kidepo Valley National Park and other national parks to determine the extent of infection and its impact on lion populations.

This is the first study of *Brucella* in Uganda kob. The percentage of positive samples in Uganda kob (11.1%) was higher than that observed in other medium sized antelopes such as impala (1.4%) in similar ranging conditions in Zimbabwe [11] and black lechwe (*Kobus leche smithemani*) (0%) in Zambia [35]. The percentage in the Uganda kob was lower than that found in Kafue lechwe (*Kobus leche kafuensis*) which was estimated at 42.9% [35]. In this case, the higher prevalence in the Kafue lechwe was related to interaction with positive cattle and infection might now be endemic within the antelope population. Positives in Uganda kob could be due to fact that Uganda kob are found in areas where they are likely to interact with livestock. Uganda kob are most likely to be taken for bushmeat. In Uganda, bushmeat consumption especially along major transit routes is becoming a serious threat to public health [22]. However, there is insufficient data on the trends of bushmeat consumption in Uganda but studies within the East African region indicating increasing incidences of bushmeat consumption in East Africa with antelopes being the most preferred source of bushmeat [36, 37]. This high percentage observed in Uganda kob could result in several human cases of infection unless mitigation measures are put in place to deter entry of bushmeat into the human food chain.

Several studies have detected positives in African giraffe populations and in this study, giraffes had the third highest percentage of positive samples of 31.7%. The percentage of positive samples in giraffes in Uganda is higher than in other African giraffe range states such as Botswana and Zimbabwe that had prevalence values of 11% and 3.7% respectively [13, 38]. Although there is no evidence of bushmeat consumption of giraffe meat in Uganda, there has been a notable increase on the number of snaring cases of giraffes in Murchison Falls National Park [39]. In the majority of the cases, snaring in Uganda is closely associated with bushmeat consumption [40].

Brucellosis has been recorded in domestic equids as far as early 1970s. Study in wild equids have been very limited [41]. In our study only one out of the 25 tested zebra was positive (4%). There have not been many studies investigating brucellosis in zebra. Assenga et al [12] found no positives in the two animals they sampled in Tanzania and Alexander et al. [13] found no positives in 21 zebras from Botswana. The only study where positives were found were in what was Rhodesia in the 1960s, where 24% of 50 tested animals were positive [42].

Recently, the Uganda Wildlife Authority launched ambitious plans to restock several protected areas with wildlife especially with those species that are threatened or pose a considerable human-wildlife conflict [43, 44]. Recently, Uganda translocated several giraffes from Murchison Falls National Park to Lake Mburo National Park and other areas [39, 45]. A study by Caron et al. [46] shows that movement of wildlife provides a conduit for the spread of disease to new susceptible populations. Therefore, interventions that involve movement of wildlife present a considerable risk of disease spread to other new areas [47]. This calls for regular screening of wildlife before undertaking translocations.

This study capitalized on using the archived wildlife samples that were already collected during the previous disease surveillance in the four major national parks. This affected the sample size and the sampling strategy that could be used to collect samples and therefore could have led to over- or under-estimating of the percentage of positive samples. Archived samples were used because the cost of sample collection in wildlife is prohibitive [48]. It involves purchase of immobilization drugs and requires experienced veterinary expertise to immobilize wildlife. Secondly, the ethical justification of the immobilization of wildlife to collect samples requires a lot of explanation because of the risk involved. Therefore, this may be one of the most available opportunities to determine percentage of positive samples in wildlife. All the national parks have some form of livestock-wildlife interaction. However, this study did not determine the level of interaction, in which location and how the interaction occurs. These reasons limited this study to use only available archived samples. Therefore, the results of the study will apply only to those parks and species sampled. In future, as samples for study of brucellosis are being collected, concurrent samples could be collected from livestock. In addition, geographical position system could be used to collect information on location and possible interaction between wildlife and livestock.

In this study, we utilized Rose Bengal plate test (RBPT) to analyze percentage of positive samples in the wildlife samples. In African buffaloes the sensitivity and specificity of RBPT has been estimated as 98.6% and 99.2% respectively [49]. However, validation in other wildlife species has not been carried out. This may have led to some false positives and false negatives. Future studies should consider using more specific and sensitive tests such as polymerase chain reaction.

## Conclusion

This study shows that the percentage of *Brucella* exposure is high in wildlife in the four major national parks of Uganda. African buffalo are the most affected species. Brucellosis has been listed among the class B bioterror agent [50] and listed also among the top five priority diseases in Uganda [51]. This study has shown that there is high percentage of *Brucella* positives in several Ugandan wildlife species and therefore, understanding the disease in wildlife will set a great foundation to its control and elimination especially at the human-livestock and wildlife interface. A lot of knowledge gaps remain in understanding the role wildlife plays in the epidemiology of brucellosis. The impact of the disease on wildlife also needs to be explored considering emerging issues such as climate change which seems to offer opportunity for emerging diseases. The impact that brucellosis has caused or is causing in wildlife in Uganda is not clear yet. What is clear is that the percentage of positive samples over the total tested is higher in wildlife than in livestock. It will be very interesting to conduct synchronized and systematic surveys for wildlife and livestock at Uganda major wildlife-livestock interface to better understand the role of wildlife and livestock in the epidemiology of brucellosis.

## Methods

### The study area

**Fig. 3:**
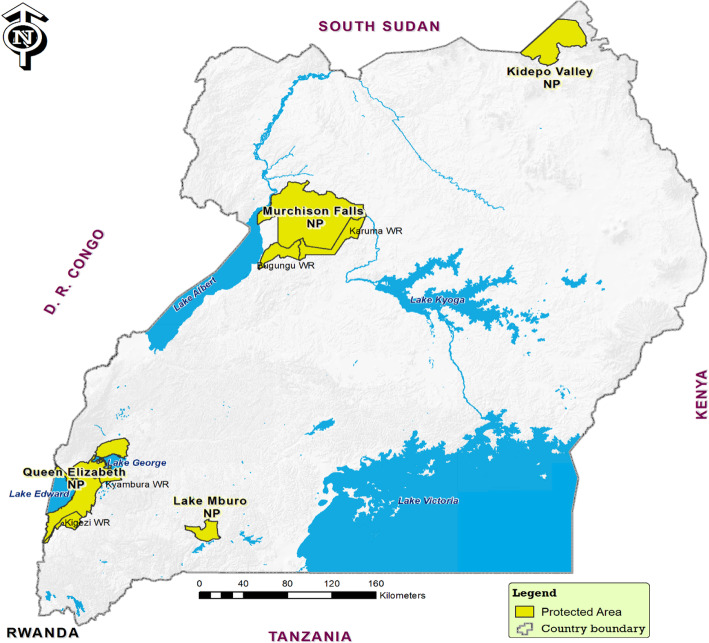
Location of National Parks in Uganda where samples were collected for Brucellosis study (Source: Authors)

Uganda is a landlocked country which lies between longitudes 4.20°N and 1.20°S, and latitude 29.5°E, and 35°W [52]. The country is in a region where seven of the distinct bioregions converge. Given the location between ecological communities between east Africa drier grasslands and west African tropical rain forests, coupled with high altitude ranges, Uganda has a high level of biodiversity [52]. Uganda has 10 national parks and 12 game reserves. This study was conducted in four major grassland national parks in Uganda, including Queen Elizabeth National Park (QENP), Lake Mburo National Park (LMNP), Murchison Falls National Park (MFNP) and Kidepo Valley National Park (KVNP) [Fig. 3]. These national parks have a high biodiversity and abundance of wildlife, and a very close wildlife-livestock interface. For instance, Lake Mburo and Queen Elizabeth National Parks have a very close wildlife-livestock interaction of the four parks selected with enclave human communities living in or around the parks with their livestock [43].

### Sample collection

This study utilized a total of 241 samples that were collected by the Uganda Wildlife Authority Veterinary Unit during routine annual surveillance in the four national parks between years 2013 and 2017. Kidepo Valley National Park contributed most of the wildlife samples used for this study. Most of the wildlife samples were from African buffalo (*Syncerus caffer*) (Table 3). Samples were also collected from African elephants (*Loxodonta africana*), giraffes (*Giraffa camelopardalis rothschildi*), Uganda kobs (*Kobus kob thomasi*), lions (*Panthera leo*), plain zebras (*Equus quagga*) and bushbucks (*Tragelaphus scriptus*).

**Table 3:** The number of different wildlife species sampled in four national parks of Uganda

National Park	No. of Samples for Each Species						
	Uganda kobs	Lions	Buffaloes	Zebras	Elephants	Giraffes	Bush- bucks
**KVNP**	0	6	24	0	0	4	0
**MFNP**	0	0	43	0	22	37	0
**QENP**	36	0	35	0	0	0	2
**LMNP**	0	0	7	25	0	0	0
**Total**	36	6	109	25	22	41	2

*KVNP* Kidepo Valley National Park, *LMNP* Lake Mburo National Park, *MFNP* Murchison Falls National Park, *QENP* Queen Elizabeth National Park

During the collection of blood samples, the wildlife were chemically immobilized following the Uganda Wildlife Authority standard veterinary protocol. Briefly, the animals to be sampled were selected randomly from a herd or group of animals. The sample size was not calculated because the population size was not known. Secondly, getting the rquired sample size reuires a significant amount of resources and lastly, the risk of an animal dying due to chemical immobilization is high. Once an animal was selected, it was immobilized using Etorphine Hydrochloride (Norvatis SA Ltd., Animal Health) at appropriate dose recommended for each species [53]. After sample collection, the effects of Etorphine were reversed by administering Diprenorphine hydrochloride (Norvatis SA Ltd., Animal Health) through the ear vein at twice the dose of Etorphine given. In the giraffes, the Etorphine effects were reversed with Naltrexone Hydrochloride at 20 mg of Etorphine used through the jugular vein [54].

In all species, 5 ml of blood were collected by venipuncture through the jugular vein. The blood in tubes was placed vertically in a rack and allowed to clot over night at room temperature. Serum was gently pipetted out into cryovials and placed in liquid nitrogen and transported to the Ministry of Agriculture, Animal Industry and Fisheries, National Animal Disease Diagnostic Centre, Entebbe, Uganda and stored at − 20 °C pending laboratory analysis. All the animals were released back into their natural habitats near where they were immobilized from.

### Laboratory analysis

The analysis was done according to the protocol for Rose Bengal plate test (RBPT) set by the World Health Organisation for Animal Health [55]. Briefly, the test procedure was as follows: the serum samples were removed from − 20 °C freezer and kept at 4 °C overnight to allow the serum to thaw. Samples were then sorted and all hemolyzed samples were removed and not included in the analysis. The sample vials were placed into a rack. Twenty-five microlitres of each sample was placed on a white tile and 25 μl of positive control added. Twenty-five microlitres of Rose Bengal reagent (Onderstepoort Biological Products Pty, South Africa) was gently added to each of the samples. The samples and the reagent were gently mixed using an applicator stick in a circular manner. The tile was rocked for 4 min at room temperature. Observation was made for agglutination within 4 min and recorded. After reading the results, the tile was washed with distilled water and dried.

### Data analysis

Initially all data was entered in Microsoft Excel v2016, sorted and checked for completeness. Data was exported to Statistical Package for the Social Sciences v20 for analysis. At bivariate level, data was summarized using frequencies and percentages. The percentage of positive samples of brucellosis was calculated by taking the number of infected animals as a percentage of total number of animals sampled per animal species and per national park. The percentage of positive samples of brucellosis was compared by species and by national park using Goodman and Kruskal’s Gamma (G-test). The difference in percentage of positive samples of brucellosis was done using multiple comparison post-hoc of proportions and the G-statistics, together with their *p*-values, were reported. All the analysis was carried out at 95% CI and the variables with *P*-Values less than 0.05 were considered significant in this analysis.

## Data Availability

The samples used during the study are available upon request to UWA and Ministry of Agriculture Animal Industry and Fisheries National Disease Diagnostic and Epidemiology Centre and the relevant government of Uganda bodies ethical approval sought. The data used for analysis is all presented here. All the animals from which the blood samples were collected were released back into the wild.

## Abbreviations

AFROHUN: Africa One Health University Network
ANOVA: Analysis of variance
COVAB: College of Veterinary Medicine, Animal Resources and Biosecurity
ILRI: International Livestock and Research Institute
KVNP: Kidepo Valley National Park
LMNP: Lake Mburo National Park
MFNP: Murchison Falls National Park
NEMA: National Environmental Management Authority
OIE: World Organization for animal health
QENP: Queen Elizabeth National Park
RBPT: Rose bengal plate test
UK: United Kingdom
UNCST: Uganda National Science and Technology
UWA: Uganda Wildlife Authority
WCS: Wildlife Conservation Society
